# Inequality constraint on the maximum genus for 3D structural compliance topology optimization

**DOI:** 10.1038/s41598-022-20248-x

**Published:** 2022-09-28

**Authors:** Haitao Han, Chong Wang, Tongxing Zuo, Zhenyu Liu

**Affiliations:** 1 Changchun Institute of Optics, Fine Mechanics and Physics (CIOMP), Chinese Academy of Sciences, Changchun, 130033 China; 2 School of Optoelectronics, University of Chinese Academy of Sciences, Beijing, 100049 China

**Keywords:** Mechanical engineering, Applied mathematics

## Abstract

Structural topology constraints in topology optimization are an important research topic. The structural topology is characterized by the topological invariance of the number of holes. The holes of a structure in 3D space can be classified as internally enclosed holes and external through-holes (or tunnels). The genus is the number of tunnels. This article proposes the quotient set design variable method (QSDV) to implement the inequality constraint on the maximum genus allowed in an optimized structure for 3D structural topology optimization. The principle of the QSDV is to classify the changing design variables according to the connectivity of the elements in a structure to obtain the quotient set and update the corresponding elements in the quotient set to meet the topological constraint. Based on the standard relaxation algorithm discrete variable topology optimization method (DVTOCRA), the effectiveness of the QSDV is illustrated in numerical examples of a 3D cantilever beam.

## Introduction

Recently, based on the description of the size, shape and number of holes of an optimized structure^1^, topology optimization has been implemented from a geometric complexity perspective in a given design domain. The number of holes in the structure is implicitly designed, and the set of elements in the holes is constrained^2–4^. In the 3D case, the number of holes is a topological invariant that is classified as the number of internal enclosed holes (or enclosed voids) and the genus (or the number of tunnels of a structure). In the study of the numerical instability of topology optimization, Sigmund et al.^5^ noted that there are infinite numbers of holes in the global optimal result. Therefore, structural complexity control is challenging and necessitates substantial theoretical research for structural topology optimization. The structural complexity control of topology optimization can be interpreted topologically to constrain the number of enclosed voids and the genus of a structure.

Methods of controlling the number of holes of a structure can currently be divided into fuzzy and precise methods. A fuzzy method is defined as one that satisfies the following two conditions: (a) neither the genus nor the number of enclosed voids in the optimized structure is considered; (b) the genus and the number of enclosed voids are handled simultaneously in the optimized structure. For example, a fuzzy method controls the number of holes without measuring the topological invariance of a structure, such as in the filter method^6–9^, size control method^10–15^, moving morphable components (MMC) method^16^, and intelligent cavity creation (ICC) method^17^. A precise method is defined as one that satisfies the following two conditions: (a) the genus or the number of enclosed voids in the optimized structure can be calculated directly; (b) the genus or the number of enclosed voids in the optimized structure can be controlled. Precise methods can be subdivided as follows: (a) an inequality constrains the number of enclosed voids; (b) an equality constrains the number of enclosed voids, such as in the application of graph theory and set theory to control the number and sizes of the enclosed voids of topologically optimized structures^2^, the virtual temperature approach^18,19^used to eliminate enclosed voids and fulfil the connectivity requirement, and the method of imposing an equality constraint on the number of enclosed voids with discrete sensitivity^20^; (c) an inequality constrains the genus^3,21^ ; (d) an equality constrains the genus; and (e) inequality or equality constraints exist for both the genus and the number of enclosed voids in the optimized structure. For the aforementioned five cases of precise methods, the research focused on the last three cases is limited.

The hole-filling method (HFM) constrains the maximum genus in an optimized structure in 2D space. It can be extended to constrain the maximum number of enclosed voids in 3D structure topology optimization. The genus calculation formula for the HFM is derived from the discretized Gauss-Bonnet formula^22^ for calculating the genus of a closed, orientable digital surface^23^ in a 3D grid space. However, when the method is expanded to 3D topology optimization, the following two difficulties are encountered: (a) The outer surface of a 3D structure should be an orientable and closed surface, and tunnels in a 3D structure may cross each other. Therefore, the division standard of a tunnel is difficult to determine. In the process of filling one tunnel in a solid structure, the other tunnel may be filled as well. (b) The discrete Gauss-Bonnet formula requires that the vertices on the outer surface of the structure be manifold points. However, a solid structure has an arbitrarily connected type in 3D structural topology optimization. To satisfy the maximum genus constraint on 3D structure topology optimization, one article proposed the QSDV method based on DVTOCRA^24^.

**Figure 1:**
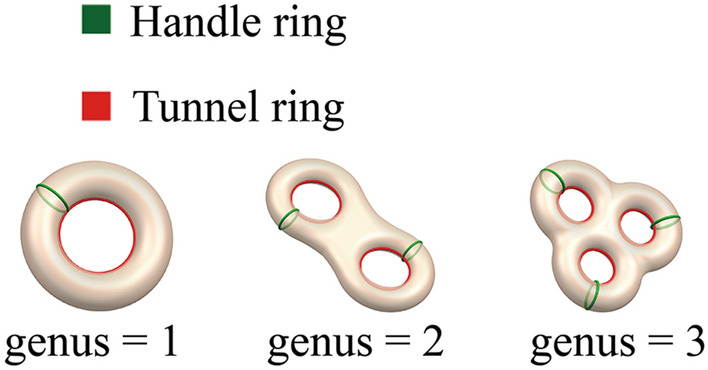
The genus corresponds to the number of handle and tunnel rings.

There are two main types of methods for calculating the genus of compact, connected, orientable, and closed surfaces: (a) direct methods such as the Gauss-Bonnet formula^25–27^ and the Euler-Poincaré characteristic number^28,29^; (b) indirect methods such as the Betti number of the surface^30–32^, the fundamental group^33^, and the first homology group of the surface^34,35^. The latter finds the bases of a tunnel. As shown in Fig. 1, a tunnel can be represented on a handle ring and a tunnel ring.

The Euler-Poincaré characteristic number that is used for calculating the genus of a 2D closed surface can be extended to the 3D structure case. It is used to construct the genus formula in this article. The calculation has high efficiency due to only counting the number of vertices, edges, faces and elements. In the numerical realization of calculating the genus, it is necessary that there be no nonmanifold vertices or edges in the structure. However, the existence of nonmanifold vertices and edges in the topology optimization process is theoretically allowed. In the numerical implementation of a nonmanifold structure, the genus calculation formula is inaccurate (for details, see Supplementary Material A). To accurately calculate the genus, a recovery geometric manifold method that converts nonmanifold vertices and edges into manifold vertices and edges is proposed.

The QSDV directly affects the volume fraction of the structure because it selects the variable update to meet the genus constraint and adds the solid elements to the recovery geometric manifold. The volume fraction of the structure cannot meet the predetermined volume constraint. A gradual removal method for structural boundary elements with structural genus invariance is proposed to meet the structural volume fraction constraint.

The QSDV needs to accurately distinguish between solid and void elements. The discrete variable topology optimization method is used for the implementation of maximum genus constraints. Discrete variable topology optimization methods include topology optimization methods based on branch-and-bound algorithms^36^ and topology optimization methods based on relaxation algorithms of integer programming^37^ and heuristic ESO^38^. DVTOCRA has not only the advantages of the SIMP method but also the advantages of the BESO method^39^. It is adopted as a platform for implementing topology constraints.

The rest of this article is organized as follows: "Quotient set design variable method" Section illustrates the theoretical background of the topology constraints of 3D structural topology optimization, the principle of the QSDV, and the principle of the evolutionary removal of structural boundary elements.  "Numerical examples" Section shows an example of topology constraints for topology optimization of a 3D cantilever beam. In "Conclusion" Section, a conclusion of the methods proposed in this article is presented.

## Quotient set design variable method

### Topology optimization model

The topology optimization problem takes the minimal compliance of the structure as the optimization objective, subject to constraints on the volume of the material, and is a standard topology optimization problem. This article focuses on the problem with an additional upper-limit constraint on the genus. Using a regular hexahedron-based mesh, the mathematical model of the topology optimization of the QSDV is described as follows:

1 $$\begin{aligned} \mathop {{\text{min}}:\,}\limits_{\rho } & c\left( \varvec{\rho } \right) = \frac{1}{2}\varvec{u}^{T} \varvec{Ku} \\ s.t.: & \sum\limits_{{i = 1}}^{N} {v_{i} \rho _{i} - \bar{V} \le 0} \\ & \varvec{Ku} = \varvec{f} \\ & \rho _{i} \in \left\{ {{\text{0}},{\text{1}}} \right\},i = {\text{1}},{\text{2}}, \cdots ,N \\ & g \le G \\ \end{aligned}$$

where $N$ is the number of elements; ${\varvec{\rho}}$ denotes the design variable vector; ${\rho }_{i}$ is a component of ${\varvec{\rho}}$; $c({\varvec{\rho}})$ is the structural compliance; ${\varvec{u}}$, ${\varvec{f}}$, and ${\varvec{K}}$ are the global displacement, external load, and structural stiffness matrix, respectively; ${v}_{i}$ is the volume of the *i*-th element; $\overline{V }$ is the prescribed volume of the material; $g$ is the genus of the structure; and $G$ is the maximum genus of the structure.

### Calculation of the genus ${\varvec{g}}$

In this article, a structure is composed of regular hexahedral elements. The structure is actually a three-dimensional CW complex. For genera in a three-dimensional CW complex, the formula below is derived (see Supplementary Material A for more details). The genus formula of a three-dimensional structure that does not contain nonmanifold vertices or edges and has no enclosed voids is

2 $${k}_{0}-{k}_{1}+{k}_{2}-{k}_{3}={c}_{n}-g$$

where ${k}_{i}$, $i=0, 1, 2, 3$, is the number of $i$-dimensional cells and ${c}_{n}$ is the number of connected components of the structure. ${c}_{n}$ can be calculated by the burning method^3^ from the number of connected components of the multiconnected structure and the element composition of each connected component.

### Recovering geometric manifold of a structure

**Figure 2:**
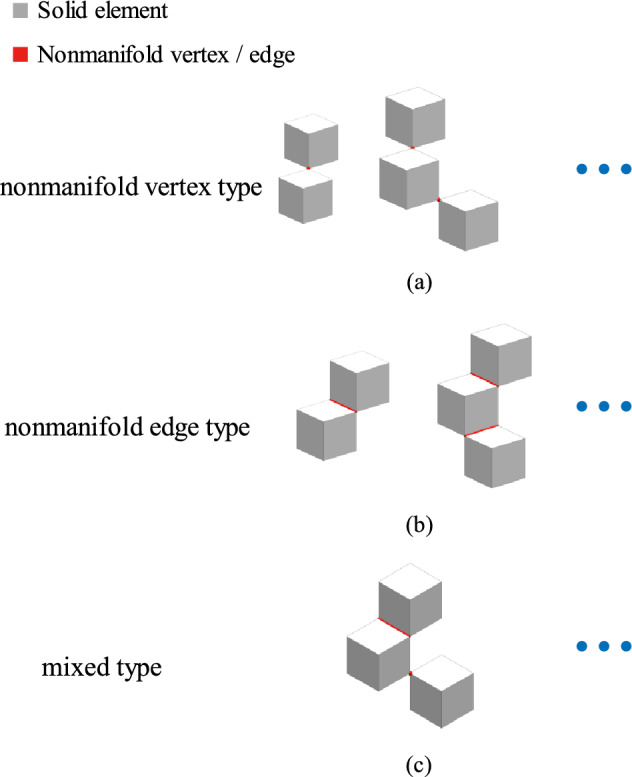
Structural representation of regular hexahedral elements.

A structure obtained in three-dimensional topology optimization may have nonmanifold vertices and edges. The structures of nonmanifold vertices and edges based on regular hexahedrons are shown in Fig. 2. According to the corresponding features, a recovery geometry manifold method that converts the nonmanifold vertices and edges into manifold vertices and edges is proposed. The core idea of the recovery geometry manifold method is to convert the void elements that connect the nonmanifold vertices and edges and have higher sensitivity into solids. First, the nonmanifold vertices and edges are identified, where a nonmanifold vertex connects only two elements and six element edges and a nonmanifold edge connects only two elements and four element faces. Second, the nonmanifold vertices are converted into manifold vertices or edges. Third, the nonmanifold edges are converted into manifold edges.

**Figure 3:**
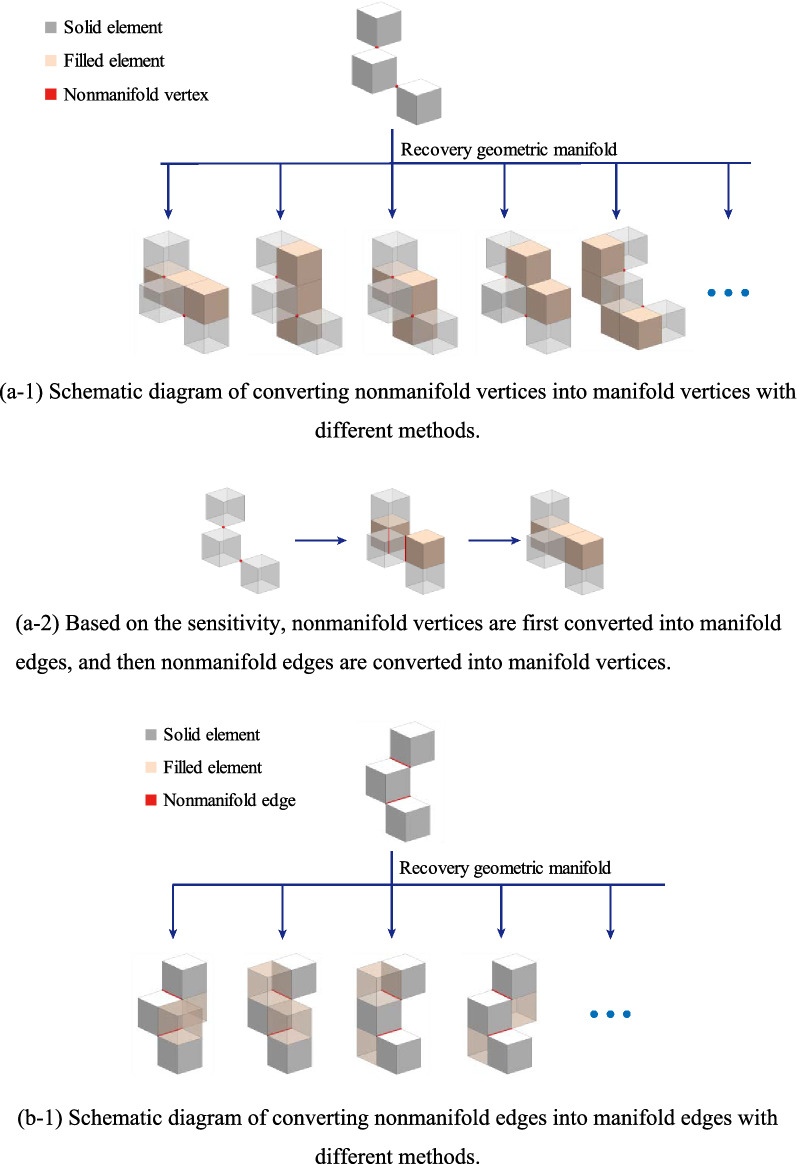

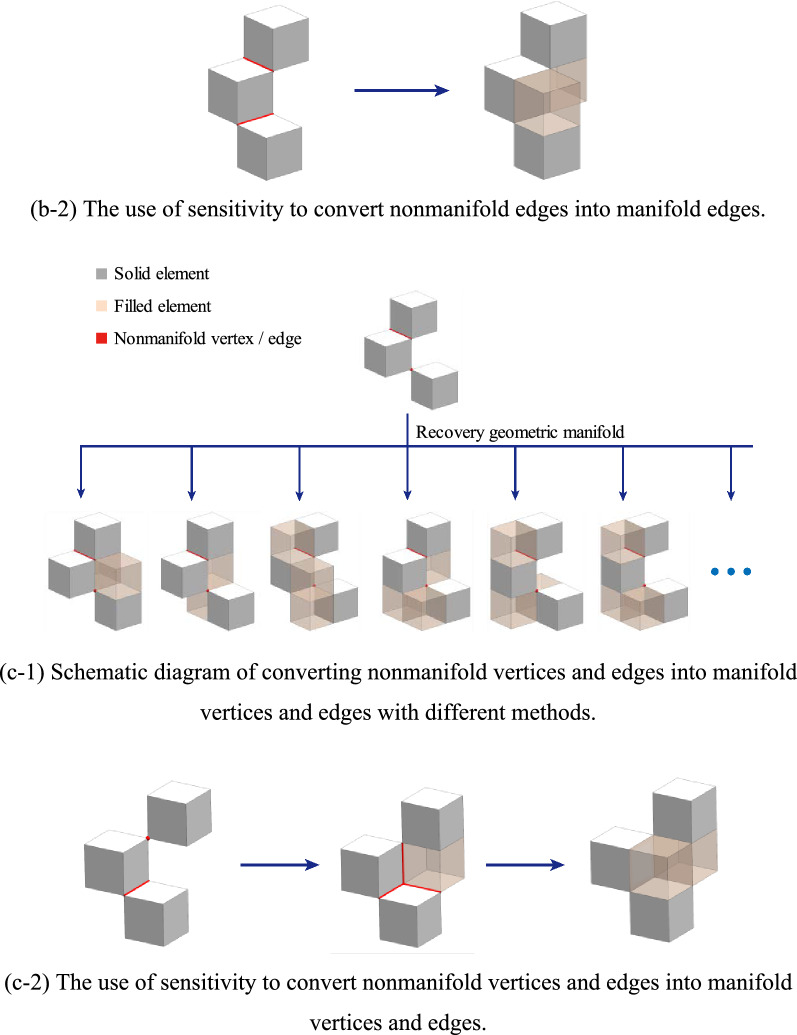
The strategy of recovering geometric manifold for nonmanifold structure.

As shown in Fig. 3, there are many strategies for the recovery geometry manifold method from a geometrical point of view.

A method based on sensitivity to first address nonmanifold vertices and then address nonmanifold edges is chosen. The process of the method is as follows:

3 $$f\left({V}_{N}\right)={V}_{fN}+{E}_{fN}$$

4 $$b\left({E}_{N}+{E}_{fN}\right)={V}_{bN}+{E}_{bN}$$

5 $$f\left({V}_{fN}+{V}_{bN}\right)={V}_{fN}+{E}_{fN}$$

6 $$b\left({E}_{fN}+{E}_{bN}\right)={V}_{bN}+{E}_{bN}$$

where $f$ is an operation that converts a nonmanifold vertex to a manifold vertex. ${V}_{N}$ is the nonmanifold vertex of the structure, ${V}_{fN}$ indicates that operation $f$ may produce a new nonmanifold vertex, ${E}_{fN}$ indicates that operation $f$ may produce a new nonmanifold edge, $b$ is an operation that converts a nonmanifold edge to a manifold edge, ${E}_{N}$ is the nonmanifold vertex of the structure, ${V}_{bN}$ indicates that operation $b$ may produce a new nonmanifold vertex, and ${E}_{bN}$ indicates that operation $b$ may produce a new nonmanifold edge.

Applying Eqs. (7) and (8) ${M}_{ve}$-1 times yields:

7 $$f\left({V}_{fN}+{V}_{bN}\right)={E}_{fN}$$

8 $$b\left({E}_{fN}+{E}_{bN}\right)=\mathrm{\varnothing }$$

Only when the operation f or operation b processes non-manifold vertex or dges at the microstructure may new non-manifold vertex or edges be formed. The filter method will filter the microstructure, and it is recommended to limit the number of microstructures by choosing a large filter radius. Thus, after a finite number of $f$ and $b$ operations, a structure is converted to a manifold structure.

### Implementation of the QSDV

The basic principle of the QSDV is to control the change in the design variables to satisfy the topology constraints on topology optimization. When the genus of the structure $s{t}_{i}$ obtained in the *i*-th iteration meets the genus constraint and the genus of the structure $s{t}_{i+1}$ obtained in the (*i* + 1)-th iteration by DVTOCRA violates the constraint of the maximum genus, the elements in the structure $s{t}_{i}$ are divided into two groups by comparison with the elements in the structure $s{t}_{i+1}$: groups of changed and unchanged elements.

The changed elements of the structure can be further divided into multiple connected subdomains. In these connected subdomains, there may be spatially symmetric connected subdomains that inherit the symmetry of the structure. This is because of the sequential updates to the design variables in the connected subdomains. The symmetry of a structure may be destroyed to meet the genus constraint. Therefore, a quotient set of the set of connected subdomains can be constructed by the geometric symmetry equivalence relationship. Finally, the elements in the quotient set are sorted by the sensitivity of the optimization objective. In descending order of total sensitivity, the elements in the quotient set that make the structure meet the genus constraint are updated.

**Figure 4:**
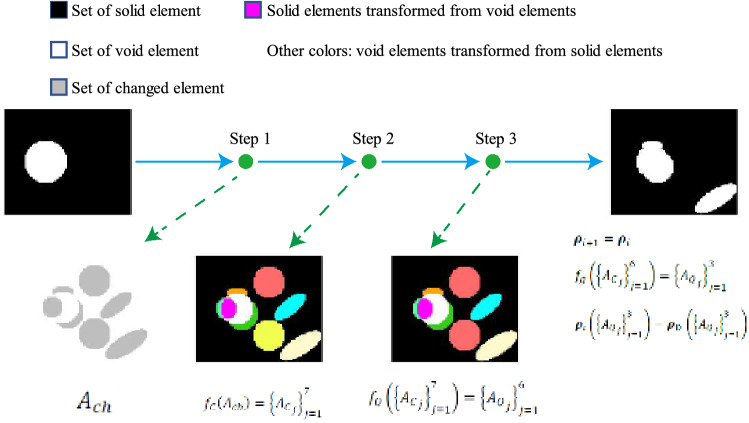
The path for the variables update in the QSDV method when the maximum genus G is assumed to be 2.

For example, the process of the QSDV method can be expressed as follows when the genus $g>G$:

9 $${A}_{ch}={A}_{i}\cup {A}_{i+1}-{A}_{i}\cap {A}_{i+1}$$

10 $${f}_{C}\left({A}_{ch}\right)={\left\{{{A}_{C}}_{j}\right\}}_{j=1}^{{C}_{n}}$$

11 $${f}_{Q}\left({\left\{{{A}_{C}}_{j}\right\}}_{j=1}^{{C}_{n}}\right)={\left\{{{A}_{Q}}_{j}\right\}}_{j=1}^{{Q}_{n}}$$

12 $${f}_{g}\left({\left\{{{A}_{C}}_{j}\right\}}_{j=1}^{{Q}_{n}}\right)={\left\{{{A}_{Q}}_{j}\right\}}_{j=1}^{{Q}_{w}}$$

13 $${{\varvec{\rho}}}_{i+1}={{\varvec{\rho}}}_{i}$$

14 $${{\varvec{\rho}}}_{i+1}\left({\left\{{{A}_{Q}}_{j}\right\}}_{j=1}^{{Q}_{w}}\right)={{\varvec{\rho}}}_{\mathrm{D }}\left({\left\{{{A}_{Q}}_{j}\right\}}_{j=1}^{{Q}_{w}}\right) \left({Q}_{w}\le {Q}_{n}\right)$$

where ${A}_{i}$ and ${A}_{i+1}$ are the solid element number sets of the i-th iteration and (*i* + 1)-th iteration, respectively, ${A}_{ch}$ is the set of changed design variables, ${f}_{C}$ is the connectivity classification operator, ${C}_{n}$ is the number of connectivity subdomains, ${f}_{Q}$ is the symmetry classification operator, ${Q}_{n}$ is the number of elements in the quotient set, ${f}_{g}$ is the selection operator for updatable elements in the quotient set, ${Q}_{w}$ is the number of updatable elements in the quotient set, ${{\varvec{\rho}}}_{i}$ and ${{\varvec{\rho}}}_{i+1}$ are the design variables of the i-th iteration and (i + 1)-th iteration, respectively, and ${{\varvec{\rho}}}_{\mathrm{D}}$ represents the design variables obtained by DVTOCRA. Figure 4 shows the path for the variables update in the QSDV method when the maximum genus G is assumed to be 2. The changed variables include those that are transformed from void elements to solid elements and from solid elements to void elements. A connected subdomain of changed variables is surrounded by black and white subdomains. Symmetrically changed variables are the same colour. The connected subdomain consists of an element set that becomes solid and an element set that becomes void, represented by two colours.

The iterative topology optimization procedure of the proposed QSDV method is described as follows:

- Discretize the design domain using a finite element mesh and assign the initial parameters for the topology optimization program.
- Perform finite element analysis (FEA), and then calculate the elemental sensitivity according to the original DVTOCRA.
- Calculate the intermediate design variables by the original DVTOCRA.
- Recover the geometric manifold of the structure.
- Calculate the genus of the structure.
- Determine whether the genus satisfies the constraint $g\le G$. If $g\le G$, proceed to step (g); otherwise, apply the QSDV method, and then proceed to step (g).
- Determine whether the volume satisfies the constraint ${\sum }_{i=1}^{N}{v}_{i}{\rho }_{i}-\overline{V }\le 0$. If ${\sum }_{i=1}^{N}{v}_{i}{\rho }_{i}-\overline{V }\le 0$, proceed to step (h); otherwise, apply the structural boundary evolution under structural genus invariance and then proceed to step (h).
- Determine whether the process has converged. If convergence occurs, proceed to step (i); otherwise, repeat steps (b)–(h) until convergence, and then proceed to step (i).
- Determine whether the stopping criteria are satisfied. If so, stop optimization; otherwise, repeat steps (b)–(i).

**Figure 5:**
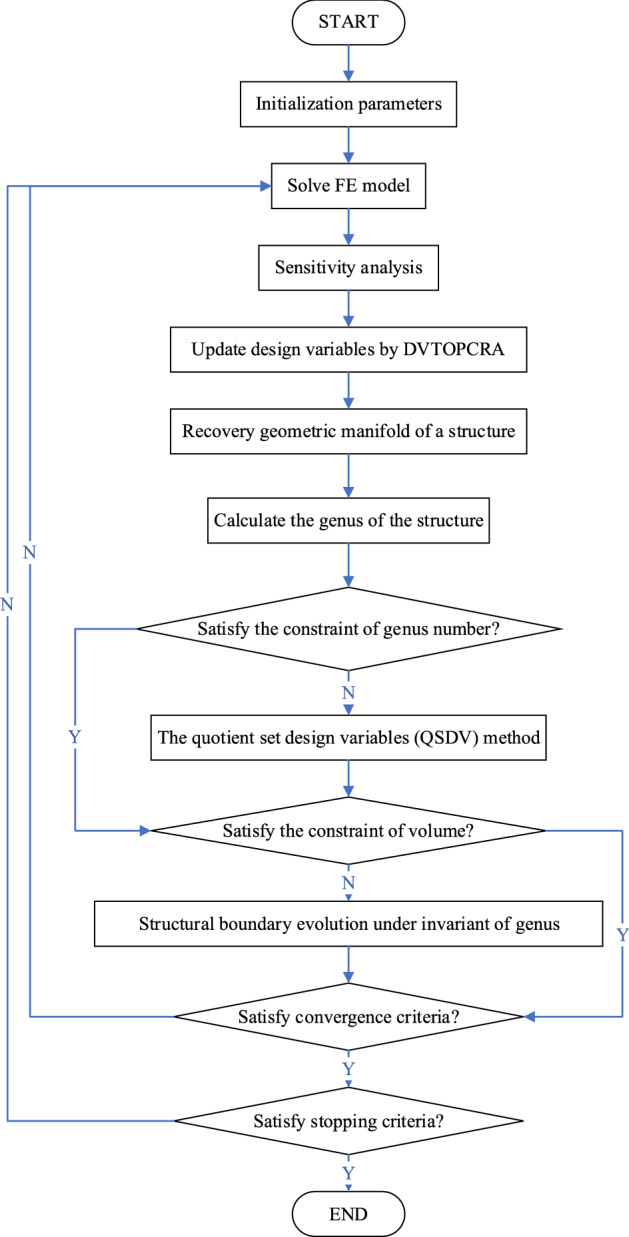
Program flow of the QSDV method based on DVTOCRA.

The program flow of the QSDV method based on DVTOCRA is shown in Fig. 5.

### Structural boundary evolution under structural genus invariance

The basic principle of the QSDV is to select some design variables to update from among the changed design variables according to the topology optimization path of DVTOCRA, while other changed variables remain unchanged to meet the structural genus constraints. The QSDV requires the initial structure to satisfy the constraint of the genus. Fortunately, because the moving limit strategy is used to meet the volume constraint, the initial volume fraction is equal to 1. When using the QSDV, the volume fraction violates the volume fraction limit predetermined by the moving limit strategy. In topology optimization, the structure obtained by the QSDV may be only slightly changed from that of the previous iteration step. Therefore, structural shape optimization is used to decrease the material volume fraction^40^.

The boundary element removal method under structural genus invariance is proposed to reduce the volume fraction of the structure based on sensitivity information. The moving limit strategy is used to control the boundary volume fraction, which is similar to ESO/BESO methods. The algorithm removes a certain number of outer boundary elements of the structure. This process is repeated until the volume fraction meets the constraint. Note that the boundary elements correspond to low-sensitivity boundary elements. In this article, the boundary element volume reduction factor is 0.99.

## Numerical examples

**Figure 6:**
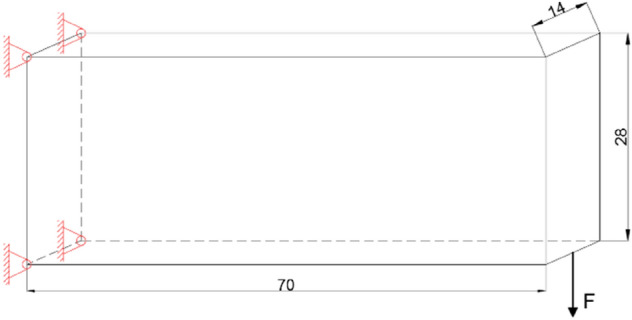
Design domain and boundary conditions for topology optimization of a three-dimensional cantilever beam.

A standard example of topology optimization of a three-dimensional cantilever beam demonstrates the effectiveness of the QSDV. The design domain is shown in Fig. 6. It is discretized into 70 × 28 × 14 regular hexahedral elements. The lower midpoint of the right end is subject to a downwards unit load.

Unless otherwise specified, the material volume fraction in this article is 0.3, the filter size is 2.1 (divided by the element size), and the volume reduction factor is 0.98.

**Figure 7:**
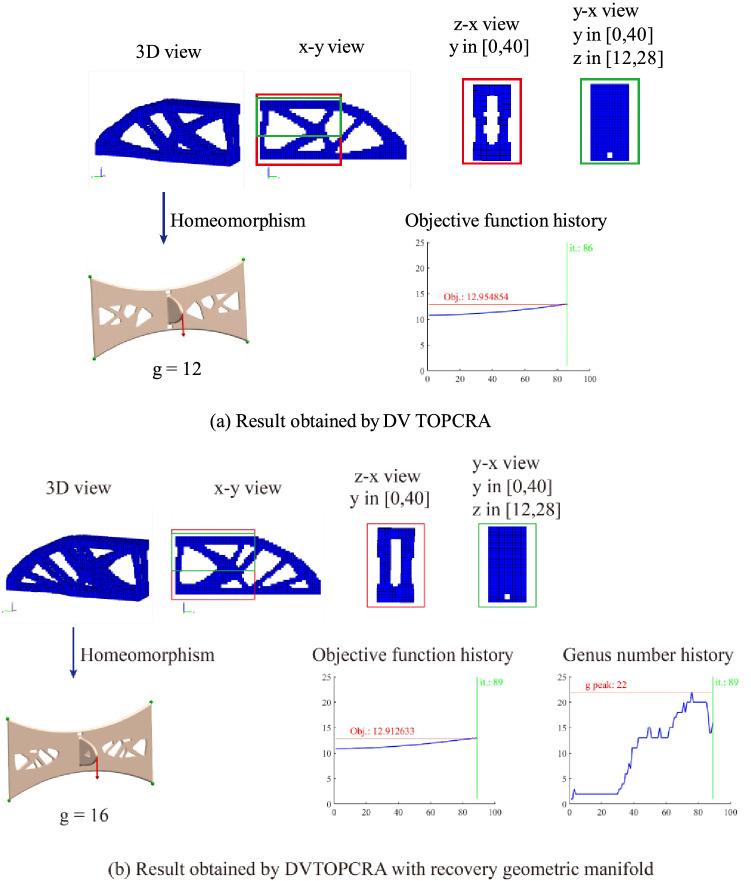
(**a**) Results obtained by DVTOCRA. (**b**) Results obtained by DVTOCRA with a recovery geometric manifold. In the homeomorphism graph, the green dots represent displacement constraints, and the red arrow represents the load.

The main reason to choose a relatively long cantilever beam is that DVTOCRA can obtain a structure with complex topology. Figure 7a shows the structure obtained by DVTOCRA. The topological homomorphic graph of the structure is given to determine the genus in the graph. The genus of the result $g$ is equal to 12, the volume fraction is 0.2999, and the objective function value is 12.954854.

The recovery geometry manifold method based on the example of Fig. 7a is added to represent the history of the genus in topology optimization and obtain the result shown in Fig. 7b. The historical variation in the genus is shown in Fig. 7b. The peak value of the genus is 22, and the genus of the result is 16. The volume fraction of the structure in Fig. 7b is 0.3004, and the objective function value is 12.912633, which is smaller than the value of 12.954854 in Fig. 7a. This might be the result of the stiffness matrix of the structure being improved by removing the nonmanifold vertices in the structure.

**Figure 8:**
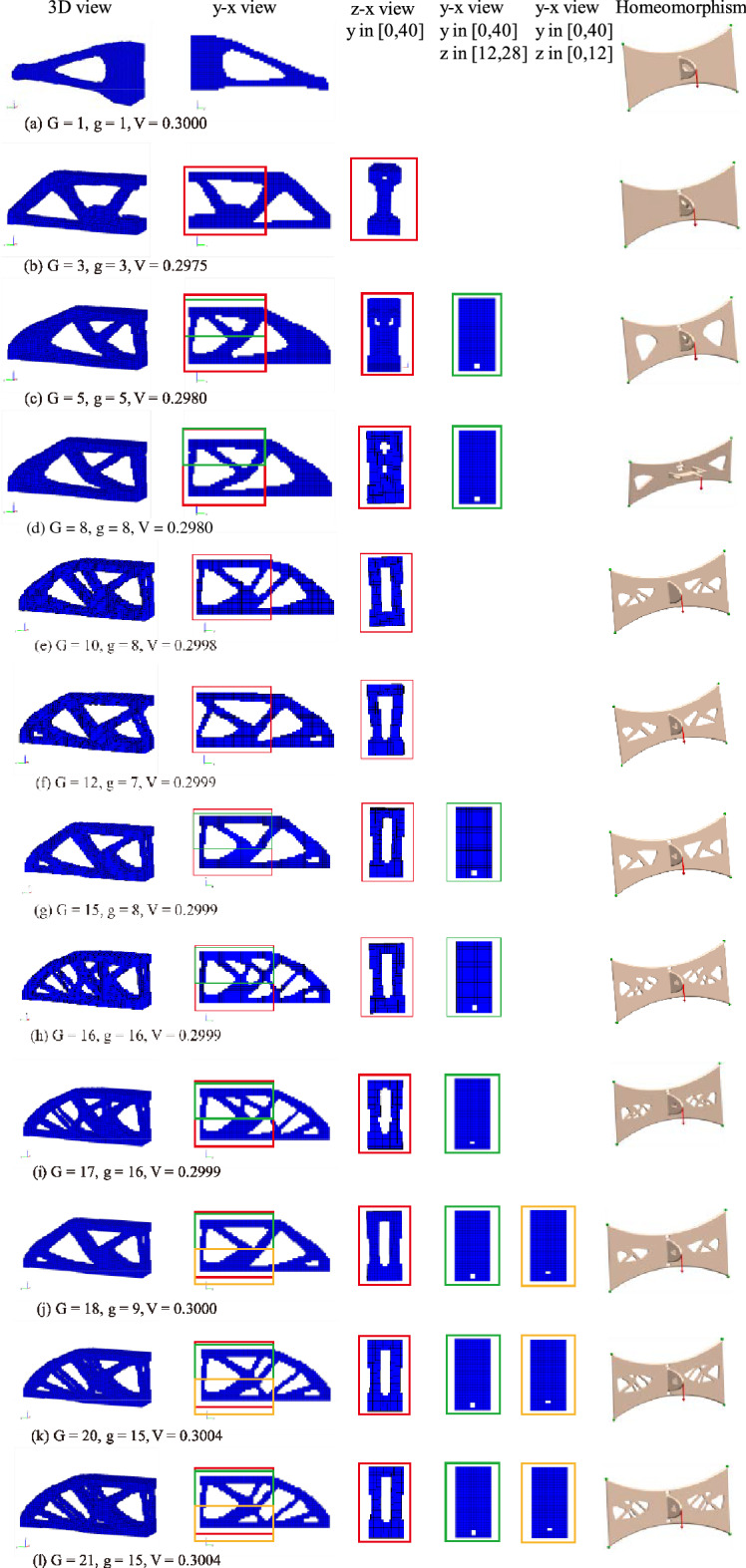
Topology constraint results of 3D cantilever beam topology optimization. G is the upper limit of the genus, and g is the actual genus of the final structure. In the homeomorphism graph, the green dots represent displacement constraints, and the red arrow represents a load.

Based on the example of Fig. 7b, topology constraints are added, and the results shown in Fig. 8 are obtained. Figure 8a–l correspond to the upper bound of the genus, with corresponding $G$ values of 1, 3, 5, 8, 10, 12, 15, 16, 17, 18, 20 and 21. In general, the genus of the result shows an upwards trend with the increase in the $G$ value. The genera of the corresponding structures are 1, 3, 5, 8, 8, 7, 8, 16, 16, 9, 15 and 15. The variation in the genus of the results also reveals that different genus constraint values $G$ have different effects on topology optimization, which is due to the different initial iteration steps of the QSDV. A greater impact on the results is correlated with a smaller value of $G$.

**Figure 9:**
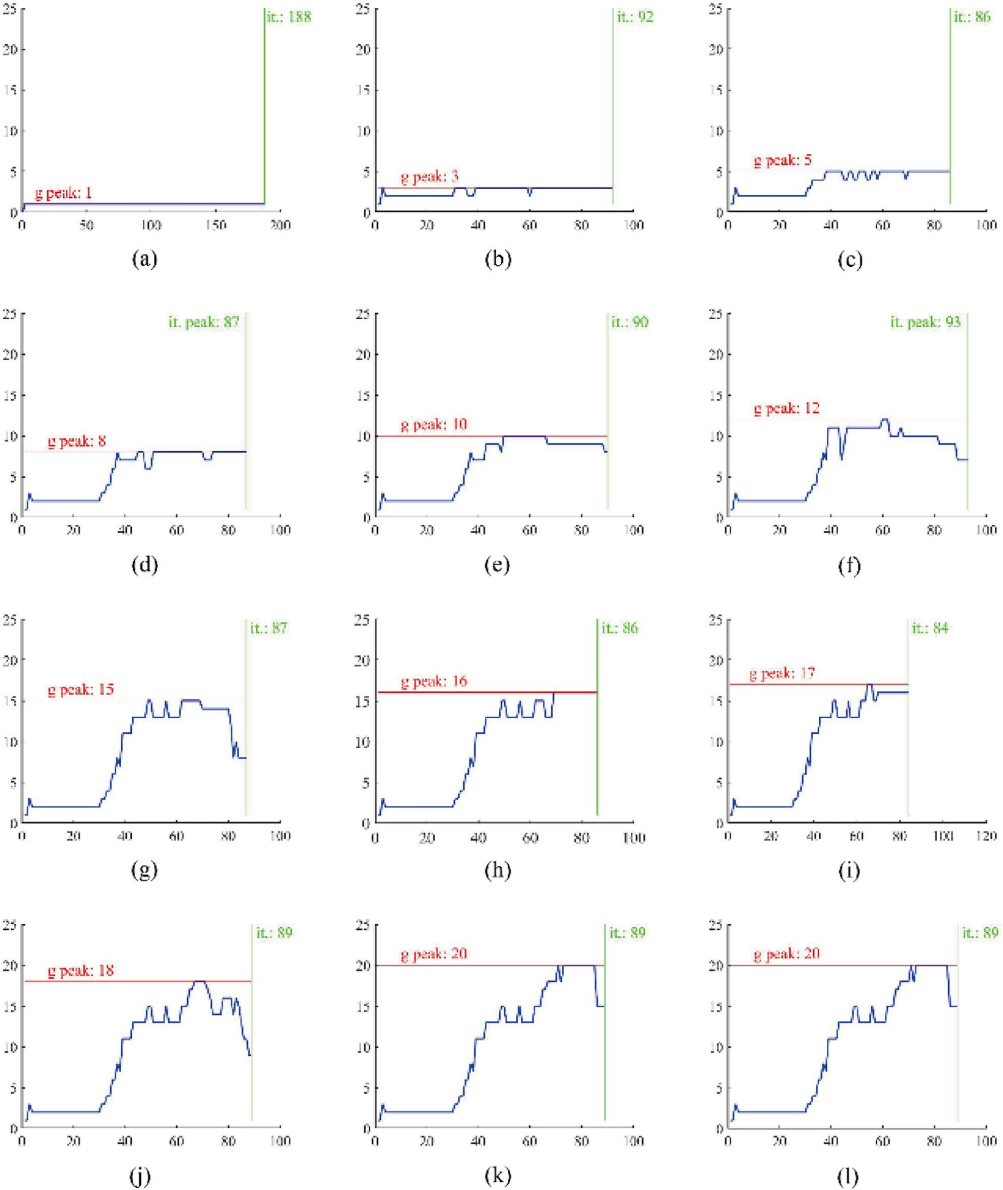
Change in the genus of the structure during the optimization process of the example in Fig. 8. The line marked “g peak” gives the maximum genus of the structure appearing in the optimization process. The line marked “it.” gives the maximum iteration step.

Figure 9 shows the change in genus in the optimization process corresponding to the example in Fig. 8. Figure 9 shows that the QSDV method can effectively constrain the maximum genus in the topology optimization. The curve of the genus parts was confirmed to be the same before the QSDV method was applied by comparing the curves of the genera in Fig. 9. This reveals that the basic principle of the QSDV method is a modification of the optimal path. The genus curves are the same when the constraint value of the genus is 20 and 21 because the influence of the connected subdomain formed by the changing element on the genus can be more than one.

**Figure 10:**
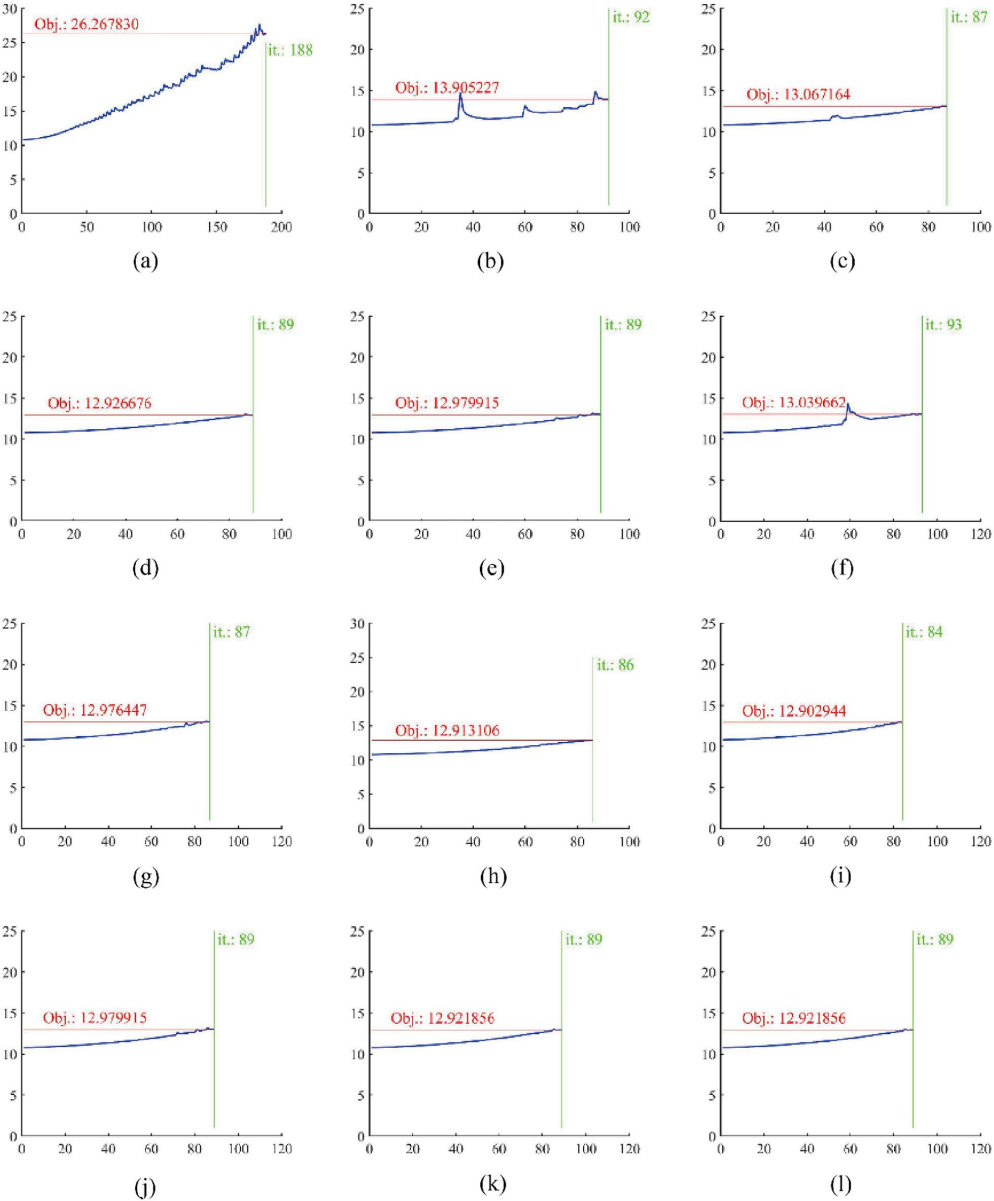
Change in the objective function value of the structure during the optimization process for the example in Fig. 8. The line marked “Obj.” gives the final value of the objective function. The line marked “it.” gives the maximum iteration step.

Figure 10 shows the change in the objective function value $c$ during the optimization process corresponding to the example in Fig. 8. The objective function value oscillates at the position where the QSDV method is applied, which means that the change in the structure topology has a notable effect on the objective function value of the structure.

## Conclusion

This article proposes the QSDV based on DVTOCRA for 3D structural topology optimization to meet the genus constraint. The essence of the QSDV is to adjust the optimized result in the topology optimization iterations to meet the constraint on the maximum genus. The numerical examples reveal that the QSDV can effectively constrain the maximum genus of an optimized structure. When the value of the upper limit G of the genus is too small, the QSDV method may converge to a local minimum solution. In such cases, one can adjust the parameters, such as the size of the filter radius, to obtain a better-performing solution.

In topology optimization, the 3D Euler-Poincaré characteristic number is used to construct the calculation genus formula. To calculate the genus of a structure correctly, a recovery structure geometry manifold is proposed based on the sensitivity of the optimal object. Using powerful tools in geometry, the combinational operations used to maintain the manifold of the surface of the optimized structure are beneficial for structural topology optimization. The implementation of the above steps is of significance for the postprocessing of geometry and subsequent computer-aided numerical analysis based on the optimized structure.

From the numerical examples, we speculate that removing nonmanifold vertices during the topology optimization process has a positive effect on the results of topology optimization. Therefore, the geometric manifold strategy has potential for further research.

## Supplementary Information

Supplementary Information.

## Data Availability

The data that support the findings of this study are available from the corresponding author upon reasonable request.
