# Supplementary material for: Inequality constraint on the maximum genus for 3D structural compliance topology optimization
Source: Sci Rep. 2022 Sep 28;12:16185. doi: 10.1038/s41598-022-20248-x (PMC9519641; doi:10.1038/s41598-022-20248-x)
Supplement: Supplementary file 1 — Supplementary Information. [file 41598_2022_20248_MOESM1_ESM.docx]

Haitao Han^ab^; Chong Wang^a^; Tongxing Zuo^ab^;Zhenyu Liu^ab^*

a Changchun Institute of Optics, Fine Mechanics and Physics (CIOMP), Chinese Academy of Sciences, Changchun 130033, China;

b School of Optoelectronics, University of Chinese Academy of Sciences, Beijing 100049, China

*Zhenyu Liu liuzy@ciomp.ac.cn

Inequality Constraint on the Maximum Genus for 3D Structural Compliance Topological Optimization

# Supplementary Material A The genus formula of a three-dimensional structure

We adopt the Euler-Poincaré characteristic number formula to derive the genus formula of the closed outer surface of a three-dimensional structure, which is well suited for topology optimization.

**Parameter Declaration**: $g$ and $g_{v}$ represent genus and the number of enclosed voids respectively.

**Definition A1.** For any finite CW complex, the Euler-Poincaré characteristic number is the alternating sum of the number of n-dimensional cells in the CW complex.

$\chi=k_{0}-k_{1}+k_{2}-k_{3}+\cdots$ (A1)

where $\chi$ is the Euler-Poincaré characteristic number, $k_{n}$ is the number of $n$-dimensional cells in the CW complex, the 0D cell is the vertex, the 1D cell is the edge, the 2D cell is the face, and the 3D cell is the element, as shown in 0.

**Definition A2.** For a finite CW complex, the maximum dimension of the cell is $n$, it is called as $n$-dimensional finite CW complex.

**Definition A3.** If in a finite CW complex, for any two cells, there exists a continuous path to connect them, it is a simply connected finite CW complex.

**Definition A4.** Let $P$ be a $k$-dimensional subcomplex of a $n$-dimensional finite CW complex, the $k$-dimensional Euler-Poincaré characteristic number is defined as $\chi_{k}\left( P \right)=k_{0}-k_{1}+k_{2}-k_{3}+\cdots+k_{k}$.

**Definition A5.** The $g$ of a three-dimensional CW complex is the number of tunnels, i.e., the number of external holes.

**Theorem A1.** (Euler formula) The Euler characteristic of an outer surface of a polyhedron is $\chi_{2}$, where $\chi_{2}=k_{0}-k_{1}+k_{2}=2$.

**Corollary A1.** If $P_{3}$ is a three-dimensional CW complex, it has $g=0$ and $g_{v}=0$. Then, $\chi_{3}\left( P_{3} \right)=1$.

Proof. The $k$-dimensional Euler-Poincaré characteristic number is a topological invariant.

Case 1: the $P_{3}$ is homeomorphic to a cube solid $C_{u}$.

Therefore, the $\chi_{3}\left( P_{3} \right)=\chi_{3}(C_{u})$.

$\chi_{3}\left( C_{u} \right)=8-12+6-1=1$ (A2)

thus, $\chi_{3}\left( P_{3} \right)=1$.

Case 2: the $P_{3}$ is not homeomorphic to a $C_{u}$.

We can assume that the $P_{3}$ consists of the cube solid. Suppose there is a nonmanifold vertex in $P_{3}$. We can divide $P_{3}$ into two parts $P_{3_{1}}$ and $P_{3_{2}}$ along the nonmanifold vertex. Thus, $P_{3_{1}}$ and $P_{3_{2}}$ are homeomorphic to a cube solid respective.

$\chi_{3}\left( P_{3_{1}} \right)=8-12+6-1=1$ (A3-1)

$\chi_{3}\left( P_{3_{2}} \right)=8-12+6-1=1$ (A3-2)

Therefore,

$\chi_{3}\left( P_{3} \right)=\chi_{3}\left( P_{3_{1}} \right)+\chi_{3}\left( P_{3_{1}} \right)-1=1$ (A4)

The number 1 is the number of the nonmanifold vertices. Then, suppose the number of nonmanifold vertices in $P_{3}$ is equal to $n_{nov}$. The $P_{3}$ can be divided into $(n_{nov}+1)$ parts along the nonmanifold vertices. Then,

$\chi_{3}\left( P_{3_{1}} \right)=8-12+6-1=1$ (A5-1)

$\chi_{3}\left( P_{3_{2}} \right)=8-12+6-1=1$ (A5-2)

$\vdots$

$\chi_{3}\left( P_{3_{1+n_{nov}}} \right)=8-12+6-1=1$ (A5-3)

Therefore,

$\chi_{3}\left( P_{3} \right)=\chi_{3}\left( P_{3_{1}} \right)+\cdots+\chi_{3}\left( P_{3_{1+n_{nov}}} \right)-n_{nov}=1$ (A6)

Suppose there is a nonmanifold edge in $P_{3}$. We can divide $P_{3}$ into two parts $P_{3_{1}}$ and $P_{3_{2}}$ along the nonmanifold edge. Thus, $P_{3_{1}}$ and $P_{3_{2}}$ are homeomorphic to a cube solid respective.

$\chi_{3}\left( P_{3_{1}} \right)=8-12+6-1=1$ (A7-1)

$\chi_{3}\left( P_{3_{2}} \right)=8-12+6-1=1$ (A7-2)

Therefore,

$\chi_{3}\left( P_{3} \right)=\chi_{3}\left( P_{3_{1}} \right)+\chi_{3}\left( P_{3_{1}} \right)-2+1$

$=\chi_{3}\left( P_{3_{1}} \right)+\chi_{3}\left( P_{3_{1}} \right)-1=1$ (A8)

The number 2 is the number of the vertices in the nonmanifold edge and the number 1 is the number of the edges in the nonmanifold edge.

Suppose the number of nonmanifold edges in $P_{3}$ is equal to $n_{noe}$. The $P_{3}$ can be divided into $(n_{noe}+1)$ parts along the nonmanifold edges. Then,

$\chi_{3}\left( P_{3_{1}} \right)=8-12+6-1=1$ (A9-1)

$\chi_{3}\left( P_{3_{2}} \right)=8-12+6-1=1$ (A9-2)

$\vdots$

$\chi_{3}\left( P_{3_{1+n_{noe}}} \right)=8-12+6-1=1$ (A9-3)

Therefore,

$\chi_{3}\left( P_{3} \right)=\chi_{3}\left( P_{3_{1}} \right)+\cdots+\chi_{3}\left( P_{3_{1+n_{noe}}} \right)-{2n}_{nov}+n_{nov}=1$ (A10)

Therefore, the Corollary A1.1 is proofed. $\square$

**Corollary A2.** If $P_{3}$ is a three-dimensional simply connected CW complex, it has $g$ and $g_{v}=0$, and there are no nonmanifold vertices and edges. Then, the Euler-Poincaré characteristic number $\chi_{3}\left( P_{3} \right)=1-g$. Moreover, if $P_{3}$ has $c_{n}$ simply connected components, then $\chi_{3}\left( P_{3} \right)=c_{n}-g$.

Proof. Suppose the structure has$g=1$, the calculation of Euler-Poincaré characteristic number of the structure as shown in 0-(a), where $M$ is a CW complex with $g=0$ and $g_{v}=0$. $Y$ is extracted from $M$ to obtain a $W$. $Y$ is a CW complex with $g=0$ and $g_{v}=0$. $W$ with $g=1$ and $g_{v}=0$. The combination of $W$ and $Y$ is $M$, and the intersecting surface of $W$ and $Y$ is $C$.

Define the following parameters: the number of vertices of $M$, $Y$, $W$ and $C$ are $k_{M0}$, $k_{Y0}$, $k_{W0}$ and $k_{C0}$ respectively. The number of edges of $M$, $Y$, $W$ and $C$ are $k_{M1}$, $k_{Y1}$, $k_{W1}$ and $k_{C1}$ respectively. The number of faces of $M$, $Y$, $W$ and $C$ is $k_{M2}$, $k_{Y2}$, $k_{W2}$ and $k_{C2}$ respectively. and the number of elements of $M$, $Y$ and $W$ is $k_{M2}$, $k_{Y2}$ and $k_{W2}$ respectively.

Obviously,

$k_{M0}-k_{M1}+k_{M2}-k_{M3}=1$ (A11)

$k_{Y0}-k_{Y1}+k_{Y2}-k_{Y3}=1$ (A12)

and,

$\left( k_{W0}-k_{W1}+k_{W2}-k_{W3} \right)+\left( k_{Y0}-k_{Y1}+k_{Y2}-k_{Y3} \right)-$

$\left( k_{C0}-k_{C1}+k_{C2} \right)=1$ (A13)

$\left( k_{W0}-k_{W1}+k_{W2}-k_{W3} \right)=(k_{C0}-k_{C1}+k_{C2})$ (A14)

$\chi_{3}\left( W \right)=\chi_{2}(C)$ (A15)

Case 1, Suppose one $Y$ corresponds to $1$ tunnels. The surface $C$ is homeomorphic to an outer surface of a polyhedron removed 2 faces. Therefore,

$\chi_{2}\left( C \right)=\chi_{2}\left( polyhedron surface \right)-2$

$=2-2=0$ (A16)

Then,

$\chi_{2}\left( C \right)=\chi_{3}\left( M \right)-1\Rightarrow\chi_{3}\left( W \right)=\chi_{3}\left( M \right)-1$ (A17)

The number 1 is the number of $Y$. Assume the tunnels in $W$ has separated from each other, then, the number of $Y$ is equal to $g$, thus,

$\chi_{3}\left( W \right)=\chi_{3}\left( M \right)-g$ (A18)

Case 2. Suppose one $Y$ corresponds to $g$.

Case 2-1. Suppose there be no nonmanifold vertices and edges in $Y$, and the outer surface of the $Y$ is homeomorphic to a polyhedron surface. Then, the $C$ is homeomorphic to an outer surface of a polyhedron removed $g+1$ disjoint faces. Therefore,

$\chi_{2}\left( C \right)=\chi_{2}\left( polyhedron surface \right)-\left( 1+g \right)$

$=1-g$

$=\chi_{3}\left( M \right)-g$ (A19)

The number $g$ represents the number of tunnels to which one Y corresponded. Therefore,

$\chi_{3}\left( W \right)=\chi_{3}\left( M \right)-g$ (A20)

Case 2-2. Suppose there be nonmanifold vertices and edges in $Y$. If there is a nonmanifold vertex in $Y$, then, The $Y$ is homeomorphic to a CW complex $P$ which consists of two cube solids connected by a vertex. Then,

$\chi_{2}\left( P \right)=2\chi_{2}\left( cube surface \right)-1=3$ (A21)

The number 1 is the number of nonmanifold vertices. If there be $n_{non}$ nonmanifold vertices in $Y$, and one of the many cases is as follows.

$\chi_{2}\left( P \right)=\left( n_{non}+1 \right)\chi_{2}\left( cube surface \right)-n_{non}$

$=\left( n_{non}+1 \right)*2-n_{non}$

$=2+n_{non}$ (A22)

The $C$ is homeomorphic to the $P$ removed $g+1$ disjoint faces. Therefore,

$\chi_{2}\left( C \right)=\chi_{2}\left( P \right)-(g+1)$

$=2+n_{non}-(g+1)$

$=1+n_{non}-g$

$= \chi_{3}\left( M \right)+n_{non}-g$ (A23)

$\chi_{3}\left( W \right)=\chi_{3}\left( M \right)+n_{non}-g$ (A24)

There are many other types for the nonmanifold vertices in $Y$ and will induce different relation equation, the article is not going to go into detail.

Case 2-3. Suppose there be nonmanifold vertices and edges in $Y$. If there is a nonmanifold edge in $Y$, then, The Y is homeomorphic to a CW complex $P$ which consists of two cube solids connected by an edge. then,

$\chi_{2}\left( P \right)=2\chi_{2}\left( cube surface \right)-2+1=3$ (A25)

The numbers 2 and 1 are the number of vertices and edges in nonmanifold edge respective. If there be $n_{noe}$ nonmanifold edges in Y, and one of the many cases is as follows.

$\chi_{2}\left( P \right)=\left( n_{noe}+1 \right)\chi_{2}\left( cube surface \right)-n_{noe}*2+n_{noe}$

$=\left( n_{non}+1 \right)*2-n_{non}$

$=2+n_{non}$ (A26)

The intersecting surface $C$ is homeomorphic to the $P$ removed $g+1$ disjoint faces. Therefore,

$\chi_{2}\left( C \right)=\chi_{2}\left( P \right)-(g+1)$

$=2+n_{noe}-(g+1)$

$=1+n_{noe}-g$

$= \chi_{3}\left( M \right)+n_{noe}-g$ (A27)

$\chi_{3}\left( W \right)=\chi_{3}\left( M \right)+n_{noe}-g$ (A28)

There are many other types for the nonmanifold edges in Y and will induce different relation equation, the article is not going to go into detail.

If the $M$ have $c_{n}$ connected components, the $\chi_{3}\left( M \right)=c_{n}$. Therefore, assume that $M$ has $g$ and $g_{v}=0$, and there are no nonmanifold vertices and edges.

$\chi_{3}\left( W \right)=\chi_{3}\left( M \right)-g=c_{n}-g$ (A29)

Therefore, the Corollary A2 is proofed. □

The Corollary A2 does not consider the case of internal enclosed holes within the structure. The tunnel and the internal enclosed holes in the structure are independent of each other, $W$ is a CW complex with g and $c_{n}$ connected subdomains.

Suppose there be no nonmanifold vertices and edges in the structure. We consider the number of the enclosed voids inside the structure.

For the case that the structure has $g_{v}=1$, the calculation of 3D Euler-Poincaré characteristic number of a structure as shown in 0-(b), where $W$ with $g=0$ and $g_{v}=0$. Extracting L from M yields the CW complex $T$. $L$with $g=0$ and $g_{v}=0$. $T$ with $g=0$ and $g_{v}=1$. The combination of $L$ and $T$ is $W$, and the intersecting surface of $L$ and $T$ is $S$.

Define the following parameters: the number of vertices of $L$, $T$ and $S$ are $k_{L0}$, $k_{T0}$ and $k_{S0}$ respectively. The number of edges of $L$, $T$ and $S$ are $k_{L1}$, $k_{T1}$ and $k_{S1}$ respectively. The number of faces of $L$, $T$ and $S$ are $k_{L2}$, $k_{T2}$ and $k_{S2}$ respectively. and the number of elements of $L$ and $T$ are $k_{L3}$ and $k_{T3}$ respectively.

Case 1, the $S$ is topologically homeomorphic to the $S^{2}$. One has,

$k_{S0}-k_{S1}+k_{S2}=2$ (A30)

$k_{W0}-k_{W1}+k_{W2}-k_{W3}$

$=\left( k_{T0}-k_{T1}+k_{T2}-k_{T3} \right)+\left( k_{L0}-k_{L1}+k_{L2}-k_{L3} \right)-\left( k_{S0}-k_{S1}+k_{S2} \right)$

$=\left( k_{T0}-k_{T1}+k_{T2}-k_{T3} \right)+1-2=1$ (A31)

and,

$\left( k_{T0}-k_{T1}+k_{T2}-k_{T3} \right)=2=1+1$ (A32)

Case 2, the S is topologically homeomorphic to the $n$-torus. One has,

$k_{S0}-k_{S1}+k_{S2}=2(1-n)$ (A33)

$k_{W0}-k_{W1}+k_{W2}-k_{W3}$

$=\left( k_{T0}-k_{T1}+k_{T2}-k_{T3} \right)+\left( k_{L0}-k_{L1}+k_{L2}-k_{L3} \right)-\left( k_{S0}-k_{S1}+k_{S2} \right)$

$=\left( k_{T0}-k_{T1}+k_{T2}-k_{T3} \right)+1-2\left( 1-n \right)=1$ (A34)

and,

$\left( k_{T0}-k_{T1}+k_{T2}-k_{T3} \right)=2(1-n)$ (A35)

If the enclosed void is not homeomorphic to $S^{2}$. The calculation of the number of the enclosed voids will become impossible. the article focuses to calculate the genus. Thus, filling the enclosed void to solid at first, and next to calculate the genus.


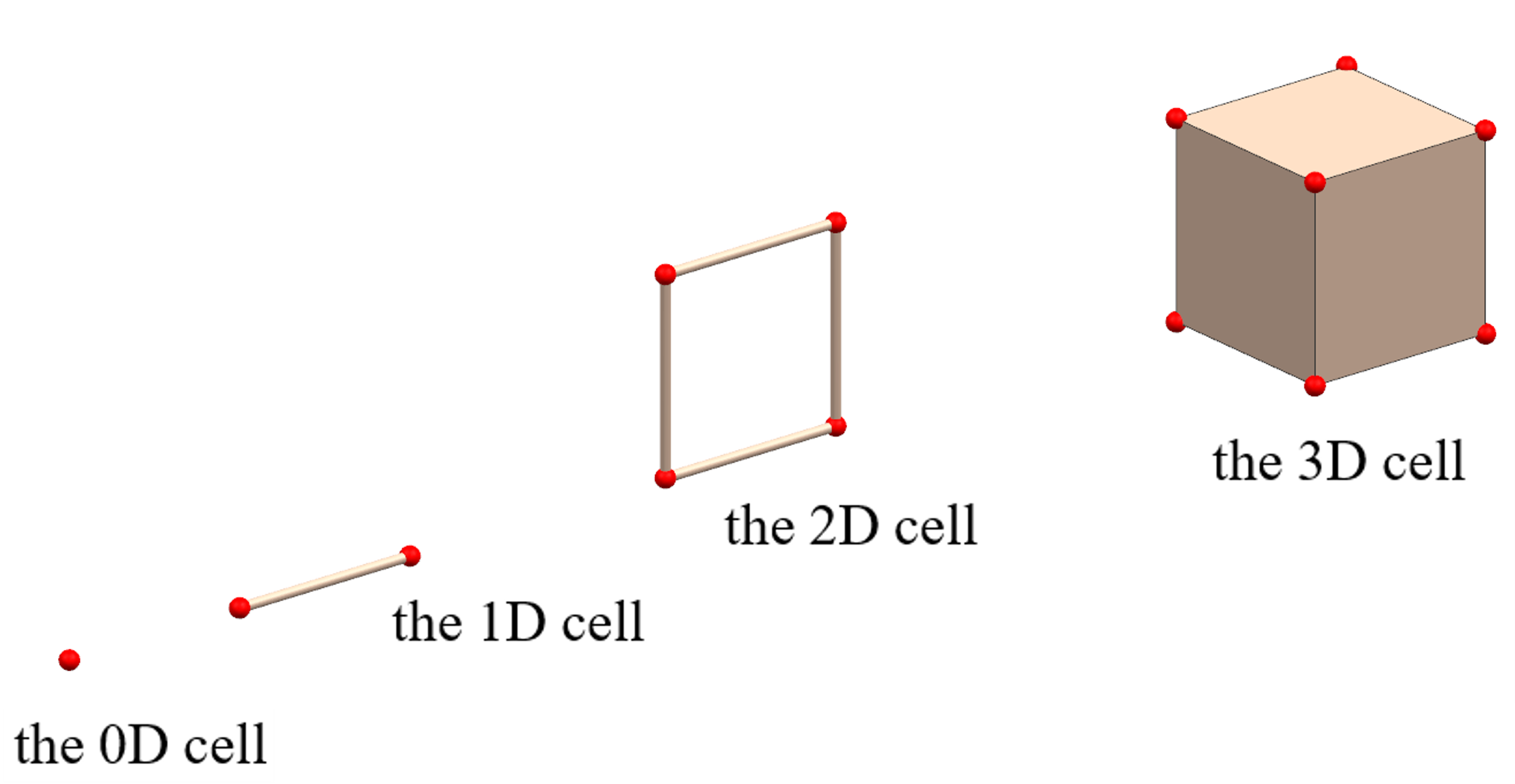


Figure A1 CW complex on different dimensions in this article: the 0D cell is the vertex, the 1D cell is the edge, the 2D cell is the face, and the 3D cell is the element.


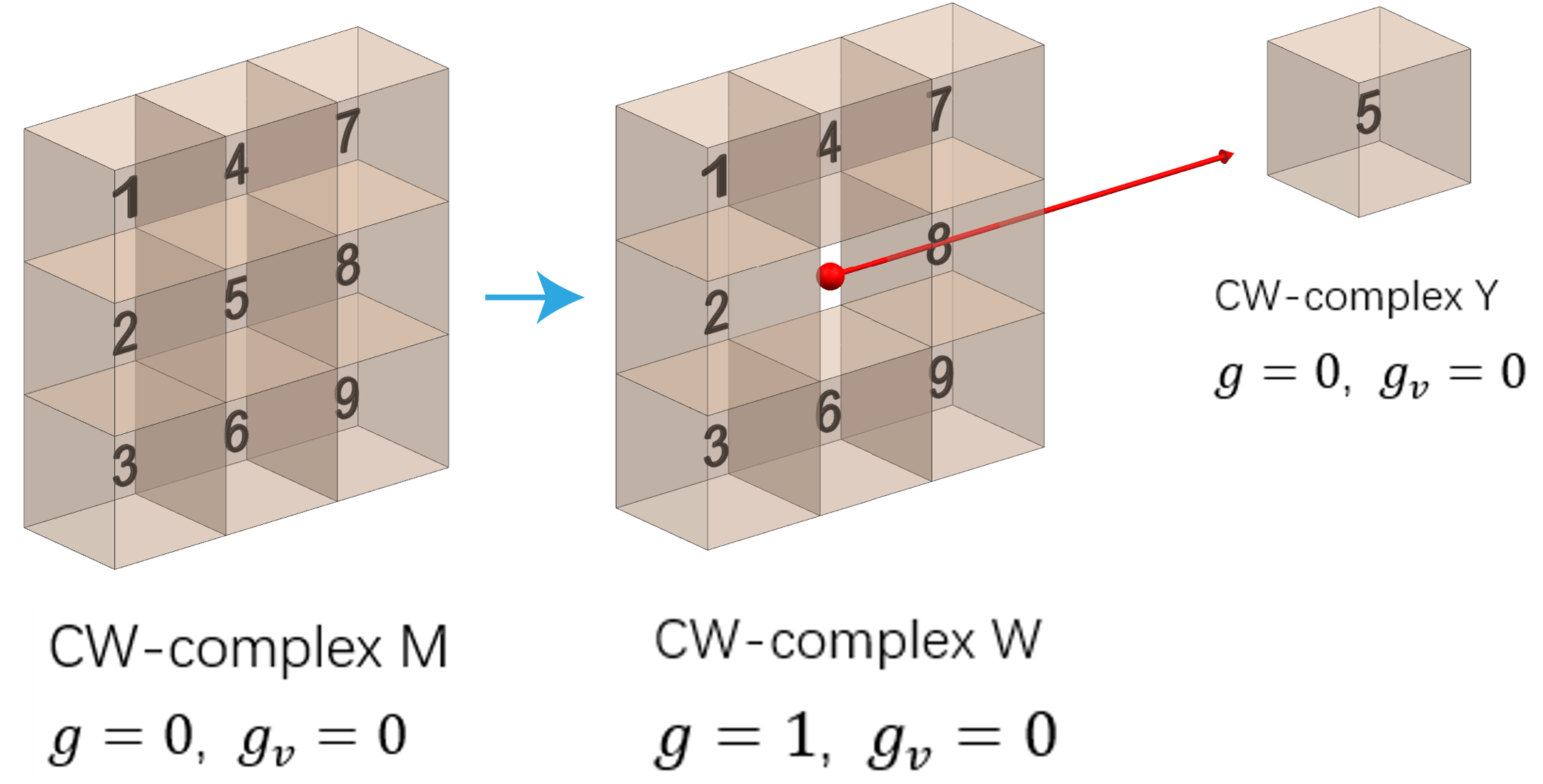


(a) To construct a tunnel in M is to dig out a CW complex Y without a tunnel and internal enclosed holes on M.


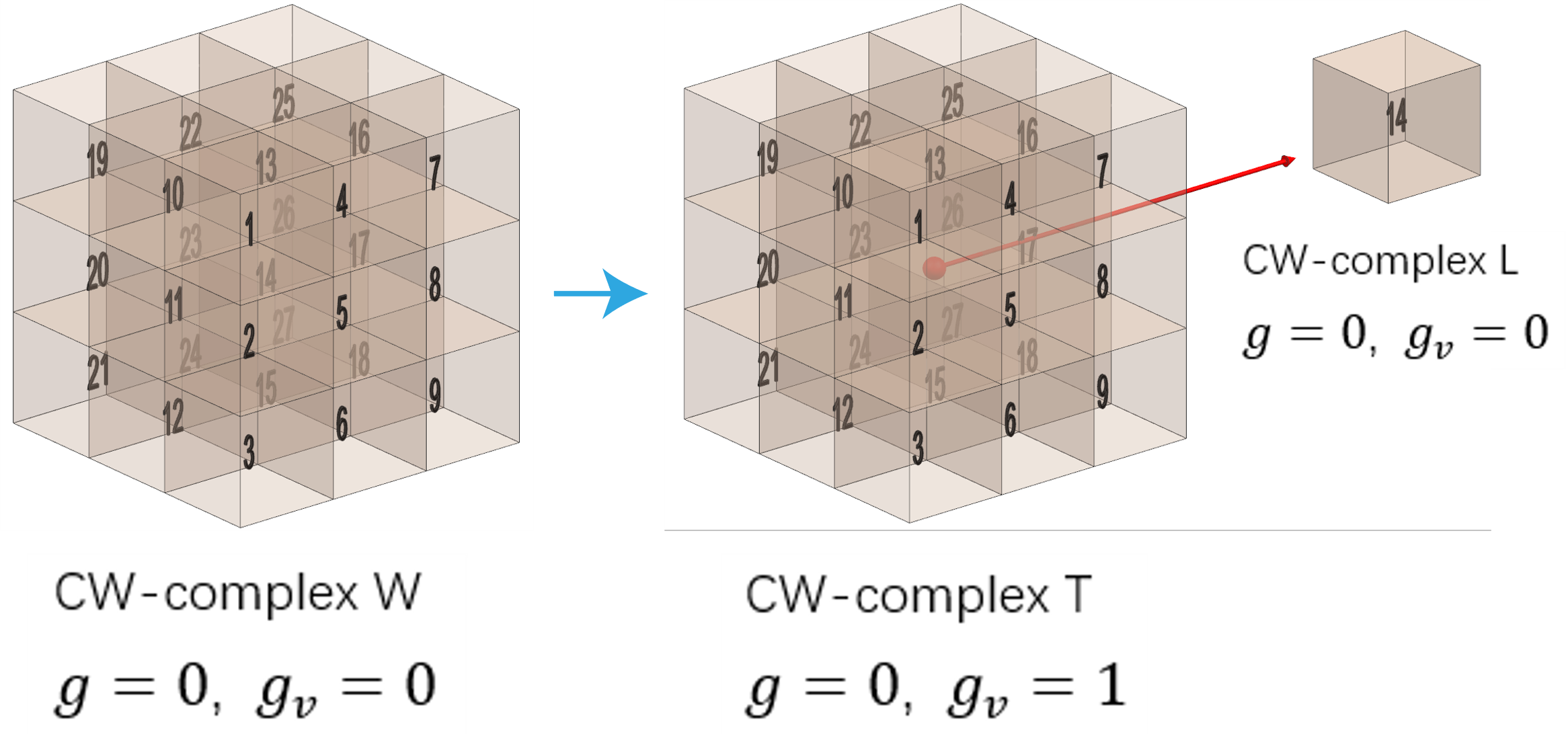


(b) To construct internal enclosed holes in W is to dig out a CW complex L without a tunnel and internal enclosed holes on W.

Figure A2 Diagram of the derivation process of the 3D Euler-Poincaré calculation genus formula. Here, $g$ is the genus, and $g_{v}$ is the number of internal enclosed holes.

# Supplementary Material B

The key formulation of DVTOCRA as below:

$$\begin{aligned} \begin{matrix} \min_{\boldsymbol{\rho}}:\quad c\left( \boldsymbol{\rho} \right)=\frac{1}{2}\boldsymbol{u}^{T}\boldsymbol{Ku} \\ \text{s.t.}:\quad\sum_{i=1}^{N} v_{i}\rho_{i}-\bar{V}\leq0 \\ \boldsymbol{Ku}=\boldsymbol{f} \\ \rho_{i}\in\left\{ 0,1 \right\}\quad i=1,2,\cdots N \end{matrix}\#\left( \text{B}1 \right) \end{aligned}$$

Here, the design domain is discretized into $N$ finite elements, and $\boldsymbol{\rho}$ denotes density design variable vector whose entry only can be zero or one. c($\boldsymbol{\rho}$) is structural compliance, $\boldsymbol{u}$, $\boldsymbol{f}$, and $\boldsymbol{K}$ are global displacement, external load, and the structural stiffness matrix, respectively. $v_{i}$ is the volume for the i-th finite element and $\bar{V}$is the prescribed the total material usage.

# Supplementary Material C

Answer:

Eq. (1) is a description of topology optimization problem, so it does not need schematic explanation.

Due to page limitations, a detailed illustration of Eq. (2) is given in appendix material B and highlight in yellow. As shown below:

$k_{0}-k_{1}+k_{2}-k_{3}=c_{n}-g$ (2)

where the $k_{i}$, $i=0, 1, 2, 3$, is the numbers of $i$-dimensional cell, the $c_{n}$ is the numbers of the connected components of structure.


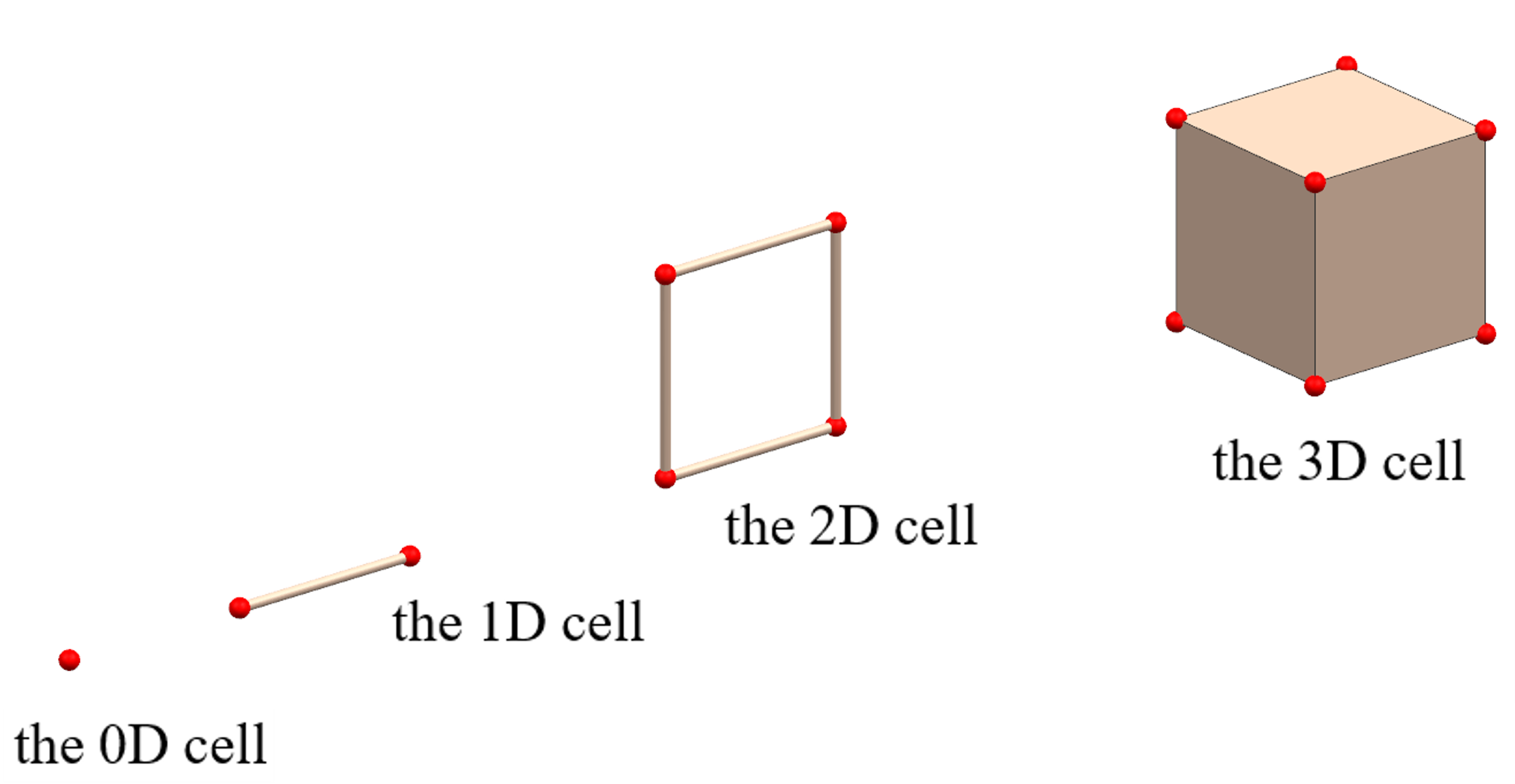


Figure A1 CW complex on different dimensions in this article: the 0D cell is the vertex, the 1D cell is the edge, the 2D cell is the face, and the 3D cell is the element.

$f\left( V_{N} \right)=V_{fN}+E_{fN}$ (3)

$b\left( E_{N}+E_{fN} \right)=V_{bN}+E_{bN}$ (4)

$f\left( V_{fN}+V_{bN} \right)=V_{fN}+E_{fN}$ (5)

$b\left( E_{fN}+E_{bN} \right)=V_{bN}+E_{bN}$ (6)

where $f$ is an operation that converts the nonmanifold vertex to the manifold vertex. $V_{N}$ is the nonmanifold vertex of structure, $V_{fN}$ indicates that operation $f$ may produce a new nonmanifold vertex, $E_{fN}$ indicates that operation $f$ may produce a new nonmanifold edge, $b$ is an operation that converts a nonmanifold edge to a manifold edge, $E_{N}$ is the nonmanifold vertex of the structure, $V_{bN}$ indicates that operation $b$ may produce a new nonmanifold vertex, and $E_{bN}$ indicates that operation $b$ may produce a new nonmanifold edge.

Appling Equations (7) and (8) $M_{ve}$-1 times in turn:

$f\left( V_{fN}+V_{bN} \right)=E_{fN}$ (7)

$b\left( E_{fN}+E_{bN} \right)=\emptyset$ (8)

Only the $f$ operation or $b$ operation is performed at the microstructure may there be a new nonmanifold vertex or edge. The filter method will filter microstructure, and it is advocated to limit the number of microstructures by choosing a large filter radius. Thus, after a finite number of $f$ and $b$ operations, a structure is converted to a manifold structure.

Eq. (3) corresponding diagram


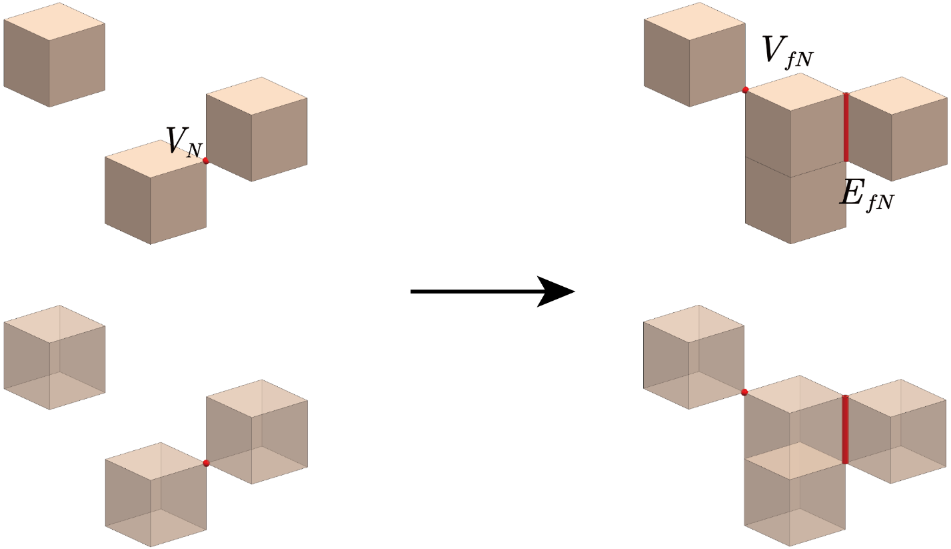


Eq. (4) corresponding diagram


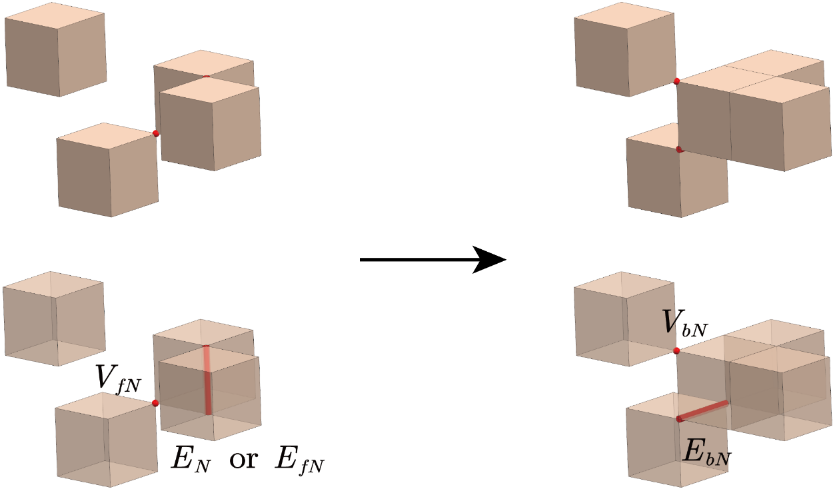


The operating principles of Eq. (5) and Eq. (6) correspond to Eq. (3) and Eq. (4) respectively.

Eq. (7) is modified, highlight it in yellow, and the corresponding diagram is:


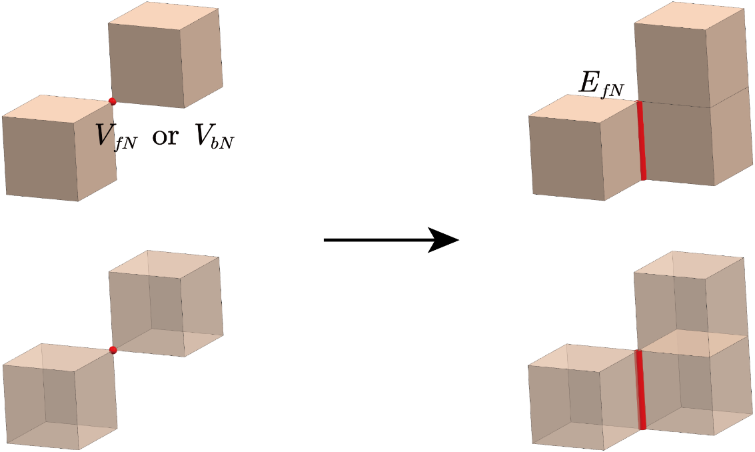


Eq. (8) corresponding diagram


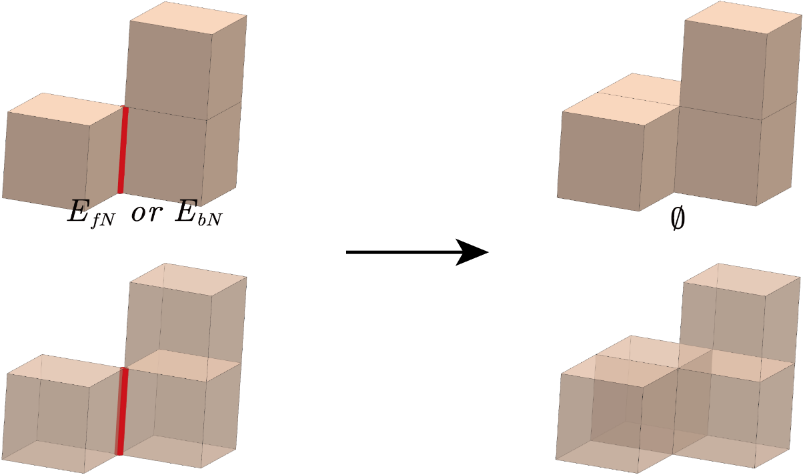


# Supplementary Material D


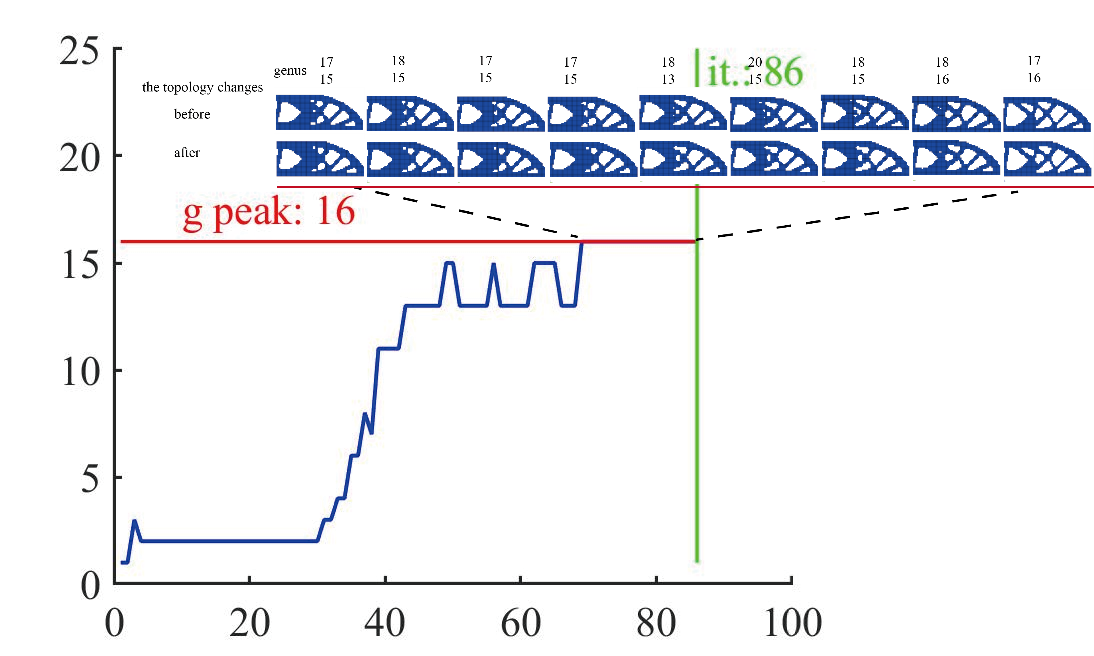


Figure The topological change of the structure corresponding to the control topology of the example whose genus is less than or equal to 16
